# Hierarchical Micro‐Nano Sheet Arrays of Nickel–Cobalt Double Hydroxides for High‐Rate Ni–Zn Batteries

**DOI:** 10.1002/advs.201802002

**Published:** 2019-02-21

**Authors:** Hao Chen, Zhehong Shen, Zhenghui Pan, Zongkui Kou, Ximeng Liu, Hong Zhang, Qilin Gu, Cao Guan, John Wang

**Affiliations:** ^1^ School of Engineering Zhejiang A&F University Hangzhou 311300 P. R. China; ^2^ Department of Materials Science and Engineering National University of Singapore Singapore 117574 Singapore; ^3^ Institute of Flexible Electronics Northwestern Polytechnical University Xi'an 710072 P. R. China

**Keywords:** hierarchical micro‐nano sheet arrays, high rate capability, nickel–cobalt double hydroxides, Ni–Zn batteries

## Abstract

The rational design of nickel‐based cathodes with highly ordered micro‐nano hierarchical architectures by a facile process is fantastic but challenging to achieve for high‐capacity and high‐rate Ni–Zn batteries. Herein, a one‐step etching–deposition–growth process is demonstrated to prepare hierarchical micro‐nano sheet arrays for Ni–Zn batteries with outstanding performance and high rate. The fabrication process is conducted at room temperature without any need of heating and stirring, and the as‐grown nickel–cobalt double hydroxide (NiCo‐DH) supported on conductive nickel substrate is endowed with a unique 3D hierarchical architecture of micro‐nano sheet arrays, which empower the effective exposure of active materials, easy electrolyte access, fast ion diffusion, and rapid electron transfer. Benefiting from these merits in combination, the NiCo‐DH electrode delivers a high specific capacity of 303.6 mAh g^−1^ and outstanding rate performance (80% retention after 20‐fold current increase), which outperforms the electrodes made of single Ni(OH)_2_ and Co(OH)_2_, and other similar materials. The NiCo‐DH electrode, when employed as the cathode for a Ni–Zn battery, demonstrates a high specific capacity of 329 mAh g^−1^. Moreover, the NiCo‐DH//Zn battery also exhibits high electrochemical energy conversion efficiency, excellent rate capability (62% retention after 30‐fold current increase), ultrafast charge characteristics, and strong tolerance to the high‐speed conversion reaction.

## 1 Introduction

The rapid emerging and development of mobile and wearable electronics, hybrid and electrical vehicles has stimulated the ever growing demand for efficient batteries with high performance and safety.^1^, ^2^, ^3^, ^4^, ^5^, ^6^, ^7^, ^8^ Indeed, over the past two decades, tremendous research efforts have been dedicated to research and development of metal‐ion batteries of high capacity and power rates,^9^, ^10^ where lithium‐ion batteries are among the most successful battery types.^11^, ^12^ However, in general, their wider applications are still severely hindered by several key performance parameters, such as the relatively low rate performance, potential safety risks, sustainability in the type of materials required for large scale uses, and environmental impact.^13^, ^14^ In contrast to the organic counterpart, aqueous rechargeable batteries employ incombustible aqueous electrolytes, which help reduce the overall cost and significantly improve the device safety, and their high ionic conductivity provides a key support to enhance the rate performance.^15^, ^16^ In particular, aqueous nickel–zinc (Ni–Zn) batteries have been attracting increasing research interests more recently, owing to their impressive theoretical specific energy density (≈372 Wh kg^−1^), relatively high output voltage (≈1.8 V), ample and low‐cost zinc resources, and low toxicity,^16^, ^17^, ^18^ thereby holding a great promise for the next generation energy storages. However, the overall practically achievable performances of Ni–Zn aqueous batteries are still far from satisfactory due to their poor specific capacity and low rate capability. One of the main bottlenecks is due to the very low capacity output reported for the cathode materials in comparison with that of metal zinc anodes (which have a high theoretical specific capacity of 820 mAh g^−1^).^16^, ^18^ Therefore, considerable research efforts are being made to extensively explore novel nickel‐based cathode materials (examples of which include oxides, hydroxides, sulfides, and phosphates), some of which are with sophistic microstructure in attempts to address one or more of the issues mentioned above.^17^, ^18^, ^19^, ^20^, ^21^, ^22^, ^23^, ^24^, ^25^, ^26^, ^27^ In comparison with single metal oxides, more active sites as well as intrinsically synergistic effects can be derived from double hydroxides. Indeed, nickel–cobalt double hydroxides (NiCo‐DHs) have been verified to possess higher capacity and thus be able to outperform the individual nickel hydroxide and cobalt hydroxide counterparts, in energy density and other performing parameters.^28^, ^29^ However, the low rate capability is still a key issue for the hydroxide‐based materials. Therefore, for high‐capacity, fast charge–discharge batteries, a key challenge is to construct rationally designed NiCo‐DH cathode with high rate capability and capacity, where an ordered micro‐nano hierarchical architecture is desired and can be successfully achieved by a facile fabrication process.

In this work, novel hierarchical structure of NiCo‐DH micro‐nano sheet arrays were rationally constructed and systematically investigated as a high‐rate cathode for Ni–Zn battery. This unique construction was successfully made through an in situ etching–deposition–growth mechanism based on the cobalt‐based metal–organic framework (Co‐MOF). The prefabricated Co‐MOF arrays supported on nickel foam purposely served as both the cobalt ion source and the skeleton template. It is the etching reaction between released H^+^ (as a result of the hydrolysis of nickel and cobalt ions) and 2‐methylimidazole (2‐MIM) linkers of Co‐MOF that induces the in situ deposition and growth of NiCo‐DH nanosheets on the microscale skeleton to create a 3D hierarchical micro‐nano sheet arrays on the Ni substrate. The whole process was facilely completed at room temperature in just one operation step without the need of heating and stirring, and thus is highly energy‐saving and of high efficiency. The as‐constructed 3D hierarchical micro‐nano sheet architecture consists of nanoscale sheets and microscale supporting skeletons, which allow an effective exposure of active materials to participate in electrochemical reactions. Both the ultrathin nanosheets and the microscale skeleton arrays are vertically aligned and exhibit appropriate interspaces, which would facilitate fast electrolyte access and ion diffusion within active materials. Moreover, the interlocked nanosheets in situ formed on the microscale skeleton are directly grown on the conductive nickel foam substrate, such that the overall structure enables an expressway for the rapid electron transfer from active materials to the current collector. Benefiting from these structural advantages in combination, the NiCo‐DH electrode obtained at 90 min of reaction time (NiCo‐90) shows a high specific capacity of 303.6 mAh g^−1^ and outstanding rate performance (80% retention after 20‐fold current increase), which remarkably surpass the levels of electrodes based on individual Ni(OH)_2_ and Co(OH)_2_, and other similar materials. The NiCo‐90, when used as the cathode in Ni–Zn battery empowers a specific capacity of 329 mAh g^−1^. Moreover, the NiCo‐90//Zn battery also showed high electrochemical energy conversion efficiency, excellent rate capability (62% retention after 30‐fold current increase), ultrafast charge, and strong tolerance to the high‐speed conversion reaction. The fabrication process for the NiCo‐90 electrode is energy‐saving, as it is done by a single step at room temperature, and greatly facilitates the large‐scale production for the next‐generation Ni–Zn batteries.

## 2 Results and Discussion

### 2.1 Morphology and Compositions

**Figure 1:**
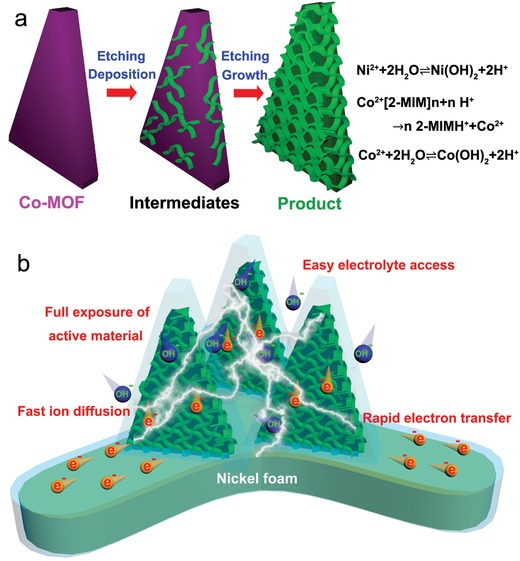
a) Formation process of NiCo‐DH hierarchical micro‐nano sheets. b) Schematic diagram of high electrochemical performance output of the as‐prepared NiCo‐DH hierarchical micro‐nano sheet arrays.

The hierarchical NiCo‐DH micro‐nano sheets were prepared by an etching–deposition–growth process as schematically depicted in **Figure** **1**a (see the Experimental Section for details). First, the preprepared Co‐MOF microsheet arrays on a Ni foam substrate was immersed into a NiSO_4_ aqueous solution at room temperature and kept stationary. The reversible hydrolysis of Ni^2+^ in near neutral aqueous solution produced a low concentration of Ni(OH)_2_ and H^+^. These H^+^ were bound to 2‐MIM linkers of Co‐MOF to form soluble 2‐MIMH^+^ in the surrounding solution, resulting in an etching reaction around the Co‐MOF skeleton to release Co^2+^.^30^ Similar to Ni^2+^, the released Co^2+^ also experienced a reversible hydrolysis to produce Co(OH)_2_ and H^+^. As the etching reaction consumed both H^+^ and 2‐MIM, the in situ hydrolysis of Ni^2+^ and Co^2+^ was promoted to form more Ni(OH)_2_ and Co(OH)_2_ leading to their codeposition on the surface of microsheet skeleton. As the process proceeded, these Ni(OH)_2_ and Co(OH)_2_ intergrew into NiCo‐DH nanosheets giving rise to a sheet‐like structure.^31^ These nanosheets in combination with the inherent microsheet skeleton led to the formation of the hierarchical micro‐nano sheet structure.

**Figure 2:**
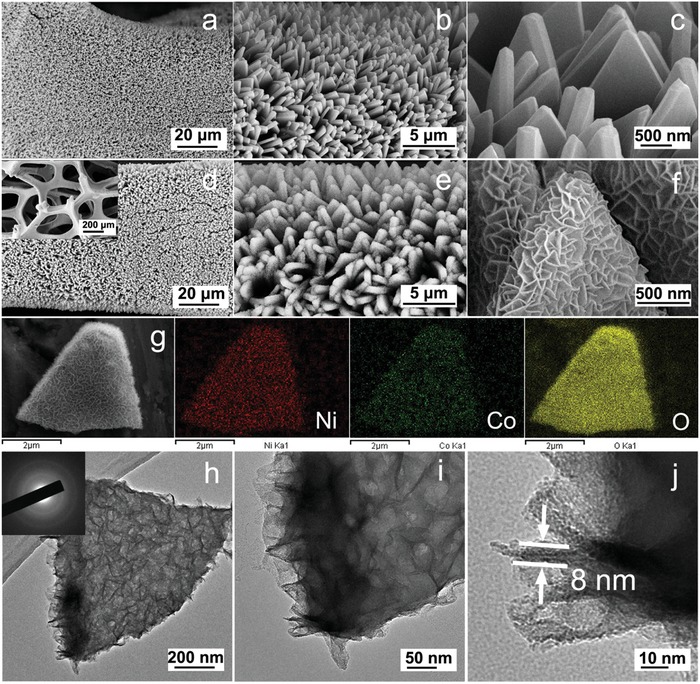
SEM images of a–c) Co‐MOF precursor, and d–f) as‐prepared NiCo‐90 supported on nickel foam. The inset is the low‐magnification SEM image of the corresponding sample. g) SEM elemental mapping, and h–j) TEM images of NiCo‐90 single microsheet. The inset is the selected area electron diffraction pattern of the corresponding sample.

As the Co‐MOF sheet‐like arrays (**Figure** **2**a–c) act as a sacrificial template for the in situ growth of NiCo‐DH, the products have inherited similar sheet‐like arrays during the whole reaction process (Figure 2d–f; Figure S1, Supporting Information). Furthermore, one can also observe that the skeleton of Co‐MOF precursor is well preserved and the interlocked nanosheets are vertically standing in the whole skeleton rather uniformly, creating abundant straight channels and pathways (Figure 2f). Scanning electron microscopy (SEM) elemental mapping images further show the uniform distribution of Ni, Co, and O elements (Figure 2g), suggesting the expected high homogeneity of nickel and cobalt hydroxides in this hierarchical micro‐nano sheet structure. To investigate further details in the microstructure, studies of transmission electron microscopy (TEM) were conducted. The as‐obtained product was observed to exhibit the hierarchical structure of nanosheet units with ultrathin feature and thickness of ≈8 nm (Figure 2h–j). Moreover, an amorphous characteristic was also identified based on the electron diffraction pattern of one whole hierarchical micro‐nano sheet (Figure 2h). These hierarchical micro‐nano sheets were vertically grown directly on the surface of conductive nickel foam, giving rise to an array structure which would facilitate electrolyte ion diffusion and electron transfer. Therefore, they would delivery outstanding electrochemical activity, when served as the battery electrode.

**Figure 3:**
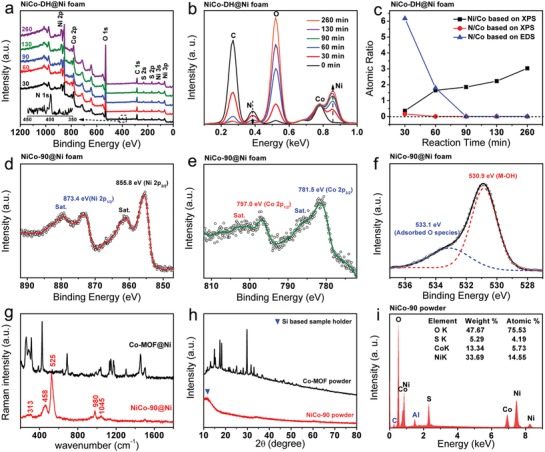
Comparisons of a) full XPS scans, b) EDS spectra, and c) atomic ratio variation of NiCo‐DHs supported on nickel foam prepared with different time. d) Ni 2p, e) Co 2p, and f) O 1s XPS scans of NiCo‐90 sample. g) Raman spectra of Co‐MOF precursor and NiCo‐90 supported on nickel foam. h) Comparisons of XRD patterns of Co‐MOF and NiCo‐90 powders. i) EDS spectrum of NiCo‐90 powder, where the Al and C signals come from the sample holder and background, respectively.

To investigate the evolution of elemental composition during the preparation process, X‐ray photoelectron spectroscopy (XPS) measurements at different reaction stages were conducted. From **Figure** **3**a, the signals of Ni, Co, and O elements are observed as expected. Moreover, it can be seen that the nitrogen element from 2‐MIM is retained after the 30 min reaction, indicating an incomplete surface etching of the Co‐MOF skeleton. After another 30 min of reaction, N signal can no longer be detected on the skeleton surface, based on the XPS full scans as shown in Figure 3a. Energy‐dispersive X‐ray spectroscopy (EDS) spectra however reveal the existence of N element in the interior of the NiCo‐60 product (Figure 3b), demonstrating that the removal of 2‐MIM was started from the outside to the inside of Co‐MOF microsheet. Based on the XPS and EDS results, the atomic ratios of Ni/Co and N/Co at various reaction stages were calculated as shown in Figure 3c. One can see that the N contents in both the surface and the interior of products drop down to zero after 90 min of reaction, manifesting a complete conversion of Co‐MOF to hydroxides. Moreover, because the solid Co‐MOF was the only source for Co^2+^ release, there was an increase tendency in the surface Ni/Co ratio over reaction time (Figure 3c), illustrating the steady codeposition and intergrowth of nickel and cobalt hydroxides. The high‐resolution Ni 2p, Co 2p, and O 1s XPS scans of sample NiCo‐90 were further investigated to identify the chemical valences of nickel and cobalt ions as well as the types of oxygen‐containing species. The Ni 2p spectrum shows two strong spin–orbit peaks at 873.4 and 855.8 eV as well as two weak satellite peaks at 879.4 and 860.9 eV (Figure 3d), respectively, which are generally believed to associate with Ni^2+^.^32^, ^33^, ^34^ Similarly, the peaks centered at 797.0 and 781.5 eV and the corresponding satellite peaks in the Co 2p spectrum can be assigned to Co^2+^ (Figure 3e).^19^ Furthermore, the O 1s XPS spectrum in Figure 3f indicates the presence of metal hydroxides and adsorbed oxygen‐containing species (e.g., H_2_O and SO_4_^2−^).^35^, ^36^ The formation of nickel–cobalt double hydroxides has been confirmed by the above XPS analysis results. In addition, Raman spectra of the precursor and NiCo‐90 product were compared to study the evolution of chemical bonds during the reaction process. Upon the etching–deposition–growth process, the resultant NiCo‐90 sample loses all Raman peaks of Co‐MOF precursor (Figure 3g), indicating a complete chemical transformation. The newly emerging peaks located at 313, 458, 525, 980, and 1045 cm^−1^ are attributed to Ni–OH/Co–OH E‐type vibration,^37^ Ni–O/Co–O bending mode,^38^ Ni–O/Co–O symmetric stretching mode,^38^ SO_4_^2−^ symmetric stretching mode,^39^ and OH deformation modes,^38^ respectively. These results again verify the formation of NiCo‐DH. Considering the strong background signal of nickel foam substrates that may seriously weaken the readability of the X‐ray diffraction (XRD), the XRD patterns of Co‐MOF and NiCo‐90 powder samples were investigated and compared to study the crystalline state of samples. As depicted in Figure 3h, the as‐prepared NiCo‐90 does not exhibit any obvious diffraction peaks except for a signal from silicon‐based sample holder, again indicating its amorphous feature, which would favour the ion penetration of aqueous electrolyte.^40^ In addition, the EDS spectra in Figure 3i shows that the molar ratio of Ni(OH)_2_ to Co(OH)_2_ in NiCo‐90 powder is near 2.5:1.

### 2.2 Electrochemical Properties of Electrodes

**Figure 4:**
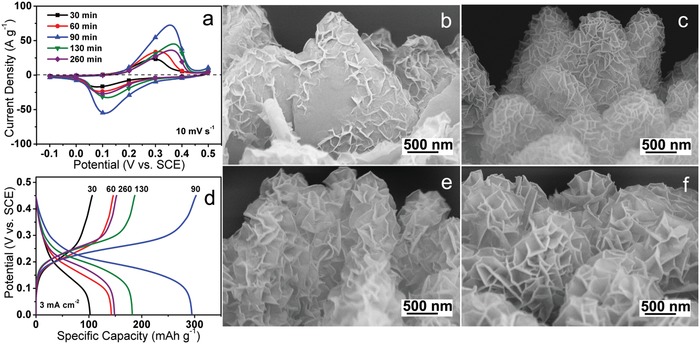
Comparisons of a) CV curves at 10 mV s^−1^, and d) CD curves at 3 mA cm^−2^ of NiCo‐DH electrodes prepared with different time. SEM images of NiCo‐DHs supported on nickel foam prepared with different reaction time: b) 30, c) 60, e) 130, and f) 260 min.

The impact of reaction time on the electrochemical performances of the as‐fabricated NiCo‐DH electrodes was first investigated in a three‐electrode system. **Figure** **4**a presents the typical cyclic voltammetry (CV) curves of NiCo‐DH electrodes prepared at various reaction times, in which a pair of redox peaks is corresponding to the reversible reactions of Ni(OH)_2_ + OH^−^ ⇌ NiOOH + H_2_O + e^−^ and Co(OH)_2_ + OH^−^ ⇌ CoOOH + H_2_O + e^−^.^19^ Because the oxidation–reduction potentials of Ni^3+^/Ni^2+^ conversion are higher than those of Co^3+^/Co^2+^ conversion,^41^, ^42^ a higher Ni/Co ratio (Figure 3c) results in the shift of CV peaks toward higher potential with the increment of reaction time. More importantly, the CV area increases first and then declines with the prolonging of treatment time, where the maximum value occurs at 90 min, suggesting that the best electrochemical activity is achieved at this time point. As expected, the charge–discharge (CD) specific capacity exhibits a similar change tendency and achieves the highest value for the electrode of NiCo‐90 (Figure 4d). This capacity variation is closely related to the morphological evolution of NiCo‐DH as displayed in Figure 4b,c,e,f. Due to the incomplete etching and deposition at 30 min, only a small amount of nanosheets was loaded on the outside of Co‐MOF (Figure 4b). XPS and EDS results have proved that there is only part of the Co‐MOF precursor converted by the reaction for 30 min. Therefore, there is limited amount of active materials for the NiCo‐30 electrode to participate in electrochemical reactions. Thus it exhibits a very weak electrochemical activity. As the synthesis time was extended to 60 min, the released Co^2+^ and surrounding Ni^2+^ hydrolyzed to produce more nickel–cobalt hydroxides nanosheets on the skeleton surface (Figure 4c). They increased the amount of active materials enhancing the electrochemical activity accordingly. With the further extension of synthesis time, the hydroxide nanosheets were more well established (Figure 2f). However, too long a reaction time caused an excessive growth of hydroxide nanosheets into large sizes and thickness (Figure 4e,f). These resulted in a reduction in the active sites per unit mass of active materials, thereby weakened the overall electrochemical activity. The NiCo‐90 electrode fabricated with a moderate treatment time of 90 min owns an explicit hierarchical micro‐nano sheet array architecture and the appropriate nanosheet size and thickness (Figure 2d–f), thereby expresses the best electrochemical performances.

**Figure 5:**
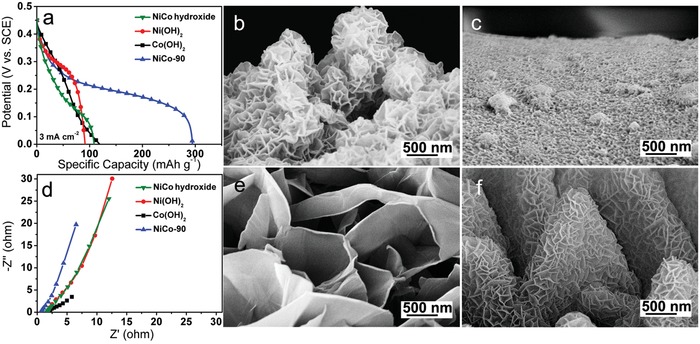
Comparisons of a) discharge curves at 3 mA cm^−2^, and d) Nyquist plots of NiCo hydroxide and Ni(OH)_2_ electrodes prepared by an electrodeposition method, pure Co(OH)_2_ electrode obtained via our etching–deposition–growth method, and NiCo‐90 electrode. Corresponding SEM images of b) NiCo hydroxide, c) Ni(OH)_2_, e) Co(OH)_2_, and f) NiCo‐90.

The optimum NiCo‐90 electrode exhibits much higher discharge specific capacity than that of the electrodes based on NiCo hydroxide and Ni(OH)_2_ synthesized by an electrodeposition method, and the Co(OH)_2_ electrode obtained via our etching–deposition–growth method, as shown in **Figure** **5**a. The comparative results of SEM images (Figure 5b,c,e,f; Figure S2, Supporting Information) reveal the superior performance of NiCo‐90 is mainly arising from the well‐defined hierarchical micro‐nano sheet array structure, in contrast to the aggregated ball‐like structure of NiCo hydroxide, the dense film of Ni(OH)_2_, and the large‐sized Co(OH)_2_ sheets (There is no Ni(OH)_2_ to depress the excessive growth of Co(OH)_2_). This well‐defined hierarchical micro‐nano sheet array structure not only supports better electrolyte ions diffusion and contact for active materials, but also promotes electron transfer from active materials to the current collector. Thus the NiCo‐90 displays a lower equivalent series resistance (*R*_s_) than the NiCo hydroxides, Ni(OH)_2_, and Co(OH)_2_ electrodes (Figure 5d). Benefitting from these features, our NiCo‐90 electrode owns the better performances, manifesting the superiority of our one‐step etching–deposition–growth process to construct the high‐performance nickel–cobalt double hydroxide electrodes.

**Figure 6:**
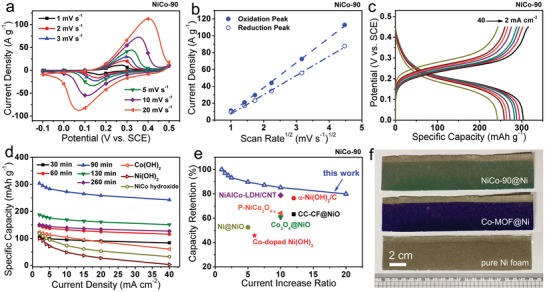
a) CV curves, b) CV peak current densities, and c) CD curves of NiCo‐90 electrodes. d) Comparison of specific capacities of Co(OH)_2_, Ni(OH)_2_, NiCo hydroxide, and different NiCo‐DH electrodes at various current densities. e) Comparison of rate capabilities of similar nickel‐ and cobalt‐based electrodes and our NiCo‐90 electrode. f) A photo displays a potential for the large‐scale production of our NiCo‐90 electrode.

In view of the remarkable electrochemical activity of NiCo‐90 electrode, its performance parameters were further investigated. **Figure** **6**a shows that the CV curve shape is well preserved with the increase in the sweep rate, and extremely high current densities are observed, implying a superior power output performance of the NiCo‐90 electrode. To further understand the electrochemical kinetics of the NiCo‐90 electrode, the CV peak current densities shown in Figure 6a were plotted against the square root of the scan rate, as presented in Figure 6b. The linear relationship between the peak current density and the square root of the scan rate demonstrates that the oxidation and reduction reactions at the NiCo‐90 electrode are diffusion‐controlled processes.^43^ In general, diffusions of electrolyte ions within the active material bulk are the rate‐limiting step for the diffusion‐controlled reaction. As shown in several previous studies, an unoptimized micro‐nanostructure can cause inadequate ion diffusion, thereby limit the capacity delivery especially at higher CD current densities. Fortunately, our NiCo‐90 electrode is capable of continuously outputting high capacity while maintaining a stable discharge platform, even at a very high current density of 40 mA cm^−2^ (Figure 6c). This is correlated to the superior morphological stability of NiCo‐90 electrode against the fast charge–discharge (Figure S3a,b, Supporting Information). By calculation based on the CD curves, the specific capacity values of NiCo‐90 electrode at different current densities are found to be much higher than those of the electrodes based on NiCo hydroxide, Ni(OH)_2_, Co(OH)_2_, and other NiCo‐DHs (Figure 6d). The maximum specific capacity of NiCo‐90 electrode can reach up to an impressive value of 303.6 mAh g^−1^ or 0.474 mAh cm^−2^ at the CD current density of 2 mA cm^−2^, and the capacity can retain 80% of its original value after a 20‐fold increasing in the CD current density, indicating an excellent rate capability of NiCo‐90 electrode. Other NiCo‐DH electrodes also exhibit superior rate performances as displayed in Figure 6d, again illustrating the superiority of the well‐constructed hierarchical micro‐nano sheet array structure in the energy delivery at high CD speeds. More importantly, the rate performances of our NiCo‐90 electrode shine among similar electrodes (Figure 6e), including for example P–NiCo_2_O_4−_ *_x_*,^18^ Co‐doped Ni(OH)_2_,^19^ Co_3_O_4_@NiO,^44^ NiAlCo‐LDH/CNT,^17^α‐Ni(OH)_2_/C,^25^ Ni@NiO,^45^ and CC‐CF@NiO.^2^ Such outstanding rate capability of NiCo‐90 electrode owe to its unique structural characteristics as described in Figure 1b. (i) The 3D hierarchical micro‐nano sheet architecture consisting of nanoscale sheets and microscale supporting skeletons allows full exposure of all active materials to participate in electrochemical reactions. (ii) The ultrathin and upright nanosheets plus the vertical microscale skeleton arrays with appropriate interspaces from hundreds of nanometers to several micrometers facilitate easy electrolyte access and fast ion diffusion within active materials.^46^ (iii) The interlocked nanosheets in situ formed on the microscale skeleton that is directly grown on the conductive nickel foam substrate, thus such structure builds up an expressway for rapid electron transfer from active materials to the current collector.^47^ These advantages have supported the capacity output under high‐speed charge and discharge conditions, thereby achieving the excellent rate performances. Based on these performances, this NiCo‐90 hierarchical micro‐nano sheet array electrode is expected as the high rate capability cathode to fabricate high‐performance alkaline batteries. In addition, the large‐size NiCo‐90 electrode can be easily prepared at room temperature without the need of heating and stirring as shown in Figure 6f, showing its great potential for the large‐scale production.

### 2.3 Electrochemical Performances of NiCo‐90//Zn Alkaline Battery

**Figure 7:**
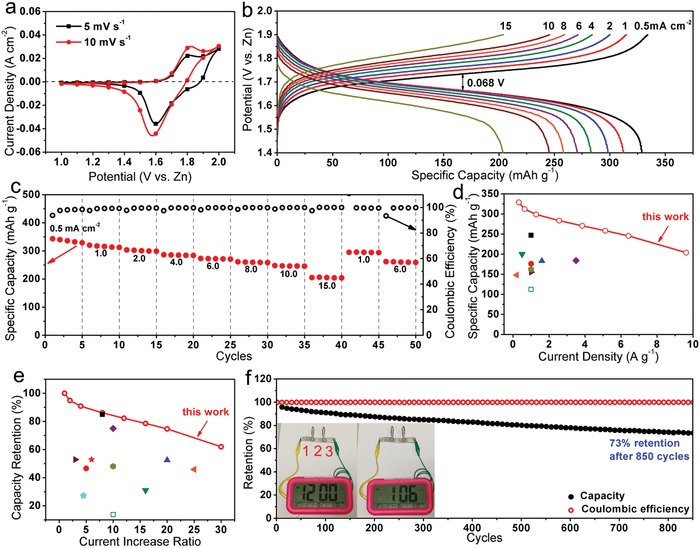
a) CV curves, b) CD curves, and c) specific capacities of as‐fabricated NiCo‐90//Zn battery. Comparisons of d) specific capacities and e) rate capabilities of the NiCo‐90//Zn battery and similar batteries reported. f) Cycling performances of this NiCo‐90//Zn battery. The inset shows that a digital clock powered by three NiCo‐90//Zn batteries connected in series.

Given the outstanding electrochemical performances of our NiCo‐90 electrode, a NiCo‐90//Zn alkaline battery was constructed with Zn plate as the anode, and researched to assess the utilization potential of this NiCo‐90 electrode as the cathode in alkaline batteries. **Figure** **7**a presents the CV curve of the NiCo‐90//Zn battery at the sweep rates of 5 mV s^−1^, which display an oxidation peak at ≈1.80 V and a reduction peak at ≈1.60 V as the response of full cell reaction^29^: Zn + NiOOH + CoOOH + 2KOH + 2H_2_O ⇌ K_2_[Zn(OH)_4_] + Ni(OH)_2_ + Co(OH)_2_. The peak positions of CV curve only has slight shifts when the sweep rate increases to 10 mV s^–1^, indicating a relatively stable electrochemical reaction for the electrical energy storage of this battery. Unlike the simple cathode reaction (only Ni^3+^/Ni^2+^conversion) in a typical Ni–Zn battery, the cathode reaction of our NiCo‐90//Zn battery involves two conversions of Ni^3+^/Ni^2+^ and Co^3+^/Co^2+^, thus the plateaus in the galvanostatic CD curves are not horizontal enough (Figure 7b). The typical charge and discharge plateaus are observed at 1.734 and 1.666 V at the current density of 0.5 mA cm^–2^, respectively, with voltage hysteresis of only ≈0.068 V. This voltage hysteresis value is much lower than the levels (0.10–0.20 V) of some typical Ni–Zn batteries reported (Table S1, Supporting Information),^2^, ^18^, ^22^, ^23^, ^24^, ^45^ indicating a less polarization feature and the high electrochemical energy conversion efficiency of our NiCo‐90//Zn battery. This point is also well supported by the high Coulombic efficiencies (near 99.5%) at various current densities in Figure 7c. Furthermore, this NiCo‐90//Zn battery exhibits impressive discharge capacities of 329, 312, 299, 283, 271, 258, 246, and 204 mAh g^−1^ recorded at current densities of 0.5, 1, 2, 4, 6, 8, 10, and 15 mA cm^–2^, respectively (Figure 7c). Based on these specific capacities, one can see that about 74.7% and 62.0% of the initial capacity could be preserved after the CD rate was elevated 20 and 30 times respectively, illustrating the excellent rate performance. At 15 mA cm^–2^, our NiCo‐90//Zn battery can be fully charged within only ≈76 s, exhibiting its ultrafast charge characteristic, compared to traditional Ni–Zn aqueous batteries which would need hours to complete the full charge. Moreover, when the discharge rate returned to 1 mA cm^–2^, the capacity recovered to 297 mAh g^−1^ (95% retention), indicating strong tolerance to the high‐speed conversion reaction. The obtainable maximum discharge capacity (329 mAh g^−1^) is much higher than those of similar Ni–Zn batteries reported (Figure 7d), such as Co‐doped Ni(OH)_2_//Zn (◼247 mAh g^−1^),^19^ NiCo_2_O_4_//Zn (▴183.1 mAh g^−1^),^20^ Co_3_O_4_//Zn (▾200 mAh g^−1^),^21^ NiAlCo‐LDH/CNT//Zn (♦184 mAh g^−1^),^17^ Ni_3_S_2_//Zn (◂148 mAh g^−1^),^22^ NiO‐CNT//Zn (▸155 mAh g^−1^),^23^ Co_3_O_4_//Zn (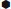
162 mAh g^−1^),^24^ NiCo_2_O_4_//Zn (⬜112 mAh g^−1^),^26^ and Ni_2_P/C//Zn (●176 mAh g^−1^).^27^ More importantly, the rate capabilities of this NiCo‐90//Zn battery clearly exceed that of a large number of similar batteries (Figure 7e), including Co‐doped Ni(OH)_2_//Zn (◼),^19^ NiCo_2_O_4_//Zn (▴),^20^ Co_3_O_4_//Zn (▾),^21^ NiAlCo‐LDH/CNT//Zn (⧫),^17^ Ni_3_S_2_//Zn (◂),^22^ NiO‐CNT//Zn (▸),^23^ Co_3_O_4_//Zn (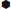),^24^ P–NiCo_2_O_4‐x_//Zn (★),^18^ α‐Ni(OH)_2_/C (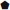),^25^ NiCo_2_O_4_//Zn (⬜),^26^ and Ni_2_P/C//Zn (●).^27^ Such superior rate performance has benefitted from the excellent electrochemical kinetics of the NiCo‐90 hierarchical micro‐nano sheet array cathode as discussed above. In addition, the long‐term cycling performances of the NiCo‐90//Zn battery were investigated at 6 mA cm^–2^. Figure 7f shows that the capacity experiences a slow decline during 850 continuous CD cycles, and then the sudden failure of the battery happened due to the fracture of zinc anode induced by the corrosion. Before the failure, near 73% initial capacity was preserved by our NiCo‐90//Zn battery, indicative of its acceptable durability. After this cycling test, the SEM morphologies of cathode and anode were also investigated as shown in Figure S3c–f in the Supporting Information. Except for minor coalescence of nanosheet units, the hierarchical micro‐nano sheet array structure of the NiCo‐90 cathode was well retained. However, a great amount of zinc dendrite was seen to form on the surface of zinc anode (Figure S3e,f, Supporting Information), which should be the main reason for the capacity decay of our battery. In addition, the NiCo‐90//Zn battery maintains a Coulombic efficiency value of above 99.5% during the whole cycling test, manifesting its excellent electrochemical reversibility. Finally, to demonstrate the potential usages of our battery in a real‐life occasion, three NiCo‐90//Zn batteries were connected in series to successfully power a digital clock (Figure 7f).

## 3 Conclusion

In this work, novel hierarchical structure of NiCo double hydroxide micro‐nano sheet arrays supported on nickel foam were facilely fabricated using a one‐step process at room temperature. The 3D hierarchical micro‐nano sheet architecture consists of nanoscale sheets and microscale supporting skeletons, which allows efficient exposure of active materials to participate in electrochemical reactions, and facilitates easy electrolyte access and fast ion diffusion within active materials. In addition, the structure is directly grown on the conductive nickel foam substrate, which enables an expressway for rapid electron transfer. Benefiting from these advantages, the as‐fabricated NiCo‐90 electrode showed a high specific capacity of 303.6 mAh g^−1^ and outstanding rate performance (80% retention after 20‐fold current increase), which remarkably outperforms the levels of electrodes based on individual Ni(OH)_2_ and Co(OH)_2_, and other similar materials. Direct usage of this NiCo‐90 electrode as the cathode in Ni–Zn battery achieved a satisfactory specific capacity of 329 mAh g^−1^. Besides, the NiCo‐90//Zn battery also exhibits high electrochemical energy conversion efficiency, excellent rate capability (62% retention after 30‐fold current increase), ultrafast charge characteristic, and strong tolerance to the high‐speed conversion reaction. These results verify that the great potential of NiCo‐90 hierarchical micro‐nano sheet array cathode in high‐rate Ni–Zn batteries. The facile fabrication process and the high electrochemical performance support the scale up for large‐scale production of the next‐generation Ni–Zn batteries. The excellent performance of our NiCo‐90 material in alkaline electrolytes makes it a valuable electrode for high‐performance supercapacitors, electrocatalyst, and biosensors.

## 4 Experimental Section

*Fabrication of Co‐MOF@Ni*: The cobalt‐based metal–organic framework supported on nickel foam (Co‐MOF@Ni) was prepared according to our previous report.^30^ In a typical experiment, the commercial nickel foam was precleaned for forming a clean surface. A 2‐methylimidazole (2‐MIM) (40 mL, 0.4 m) aqueous solution was quickly poured into the Co(NO_3_)_2_ · 6H_2_O (40 mL, 0.05 m) aqueous solution, then the clean nickel foam substrates (1 × 4 × 0.1 cm^3^, the upper 1 cm^2^ area was protected by the scotch tape) were immersed into this mixture for 4 h to grow Co‐MOF under room temperature. After the reaction, the as‐obtained sample was washed with deionized water, and then dried at 55 °C oven to obtain Co‐MOF@Ni.

*Preparation of NiCo‐DH@Ni*: The as‐fabricated Co‐MOF@Ni was immersed into a NiSO_4_ (40 mL, 16 mm) aqueous solution and kept stationary to allow an in situ conversion from Co‐MOF to nickel cobalt double hydroxide composite (NiCo‐DH) on the surface of Ni substrate. After reaction for 90 min at room temperature (≈25 °C), the resulting product was washed with deionized water for at least three times, and then dried at 55 °C to provide a NiCo‐90@Ni sample. Similarly, the NiCo‐*x*@Ni samples obtained with different reaction time (*x*) were also prepared by utilizing the similar process. For comparison purposes, the pure Co(OH)_2_@Ni was prepared by replacing NiSO_4_ solution with CoSO_4_ solution via a similar method. By following a previous report,^48^ the Ni(OH)_2_@Ni and NiCo hydroxide@Ni were fabricated by electrodepositing at −1.1 V versus saturated calomel electrode (SCE) for 30 min in the Ni(NO_3_)_2_ solution (100 mL, 50 mm) and the mixed solvent (100 mL) of Ni(NO_3_)_2_ (50 mm) and Co(NO_3_)_2_ (20 mm), respectively, followed by washing and drying the product via the similar process.

*Characterization*: The microscopic morphologies were studied using the SEM (Zeiss Supra 40) and TEM (JEOL‐2100F). An X‐ray diffractometer (Bruker D8) was employed to record the XRD patterns. The elemental compositions were investigated using XPS (Kratos Analytical Axis UltraDLD UHV) and EDS (Zeiss Supra 40) tests. The Raman spectra were collected utilizing a Raman microspectrometer (Horiba MicroRaman HR).

*Electrochemical Analysis*: The as‐prepared NiCo‐DH@Ni, Co(OH)_2_@Ni, Ni(OH)_2_@Ni, and NiCo hydroxide@Ni samples directly served as self‐supported electrodes in the electrochemical tests. Their performances were firstly researched at 25 °C in 2.5 m KOH electrolyte (saturated with ZnO) through a typical three‐electrode system using SCE and platinum (Pt) foil electrodes as the reference and counter electrodes, respectively. The electrochemical properties of a Ni–Zn alkaline battery was investigated in 2.5 m KOH electrolyte (saturated with ZnO) by employing a two‐electrode system with NiCo‐DH@Ni and a Zn foil as the cathode and anode, respectively. A Solartron 1470E electrochemical workstation (Solartron Analytical, UK) was employed to test cyclic voltammetry (CV) curves and electrochemical impedance spectra (EIS). The test of galvanostatic CD curves was completed on a Neware battery testing system (Neware, PR China). The specific capacities of the electrodes and battery were calculated from the discharge curves, according to the equation of *C* = (*I* × Δ*t*)/*m*, where *C* is the mass specific capacity (mAh g^–1^), *I* is the discharge current (mA), Δ*t* is the discharge time (h), and *m* is the mass (g) of the active material in electrodes or cathode (for the battery). The capacity retentions of electrodes and battery were calculated by dividing the specific capacity values at different current densities by the highest specific capacity value. The current increase ratios were determined by dividing the corresponding current density value by the practical current density used to obtain the highest specific capacity.

## Conflict of Interest

The authors declare no conflict of interest.

## Supporting information

Supplementary
