# Supplementary material for: Hierarchical Micro‐Nano Sheet Arrays of Nickel–Cobalt Double Hydroxides for High‐Rate Ni–Zn Batteries
Source: Adv Sci (Weinh). 2019 Feb 21;6(8):1802002. doi: 10.1002/advs.201802002 (PMC6468968; doi:10.1002/advs.201802002)
Supplement: Supplementary file 1 — Supplementary [file ADVS-6-1802002-s001.pdf]

Copyright WILEY-VCH Verlag GmbH & Co. KGaA, 69469 Weinheim, Germany, 2019.

ADVANCED SCIENCE
Open Access

Supporting Information

for *Adv. Sci.,* DOI: 10.1002/advs.201802002

Hierarchical Micro-Nano Sheet Arrays of Nickel–Cobalt Double Hydroxides for High-Rate Ni–Zn Batteries

Hao Chen, Zhehong Shen, Zhenghui Pan, Zongkui Kou, Ximeng Liu, Hong Zhang, Qilin Gu, Cao Guan,* and John Wang**

Copyright WILEY-VCH Verlag GmbH & Co. KGaA, 69469 Weinheim, Germany, 2018.

Supporting Information

Hierarchical Micro-nano Sheet Arrays of Nickel-Cobalt Double Hydroxides for High-Rate Ni-Zn Batteries

By Hao Chen, Zhehong Shen, Zhenghui Pan, Zongkui Kou, Ximeng Liu, Hong Zhang, Qilin Gu, Cao Guan*, John Wang**

Dr. H. Chen, Prof. Z.H. Shen

School of Engineering, Zhejiang A&F University, Hangzhou 311300, PR China.

E-mail: haochen@zafu.edu.cn (H. Chen)

Dr. H. Chen, Dr. Z.H. Pan, Dr. Z.K. Kou, Mr. X.M. Liu, Mr. H. Zhang, Dr. Q.L. Gu, Prof. J. Wang

Department of Materials Science and Engineering, National University of Singapore, 117574 Singapore

E-mail: msewangj@nus.edu.sg (J. Wang)

Prof. C. Guan

Institute of Flexible Electronics, Northwestern Polytechnical University, Xi'an 710072, PR China.

E-mail: iamcguan@nwpu.edu.cn (C. Guan)

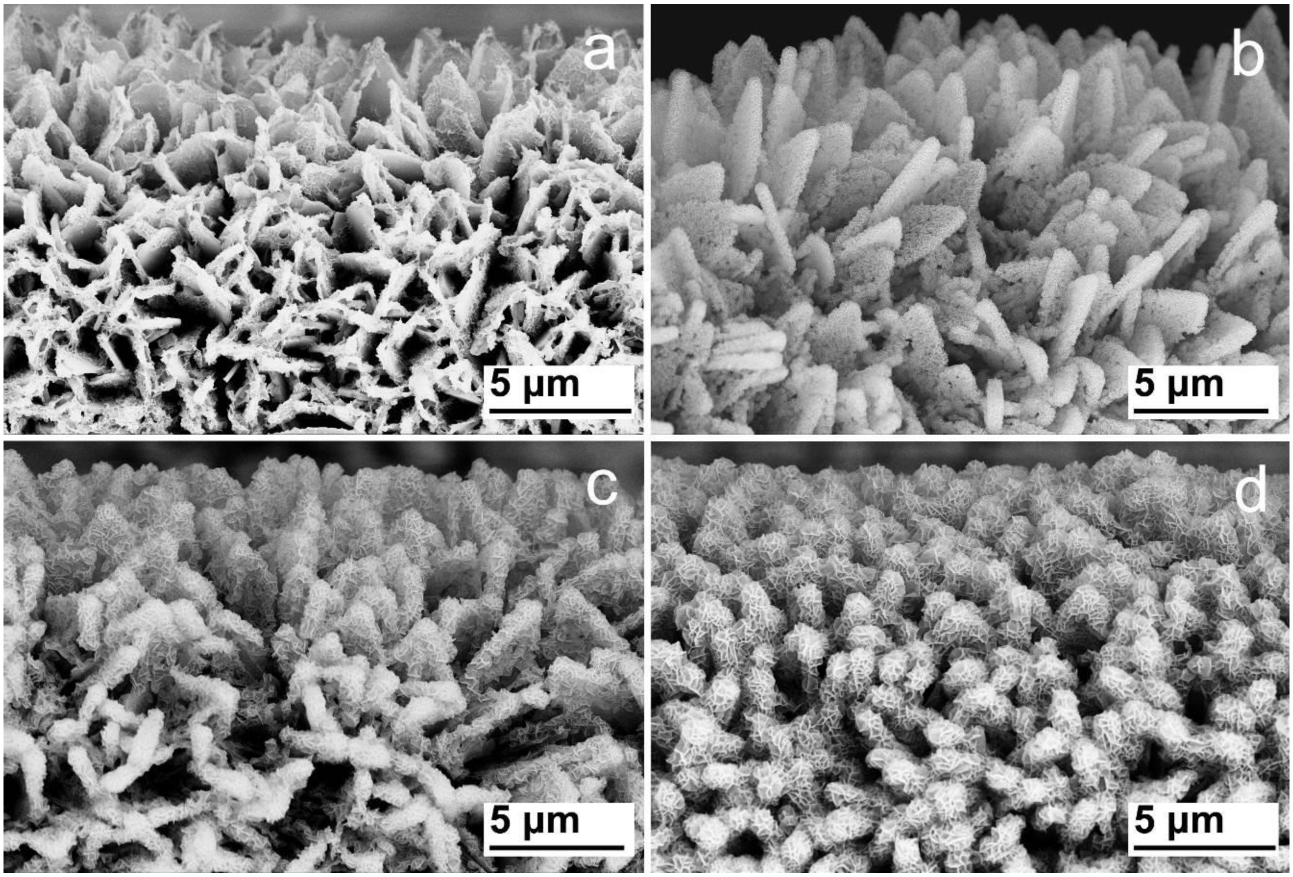

Figure S1. Comparisons of SEM images of NiCo-DHs supported on nickel foam prepared with different reaction time: (a) 30, (b) 60, (c) 130, and (d) 260 min.

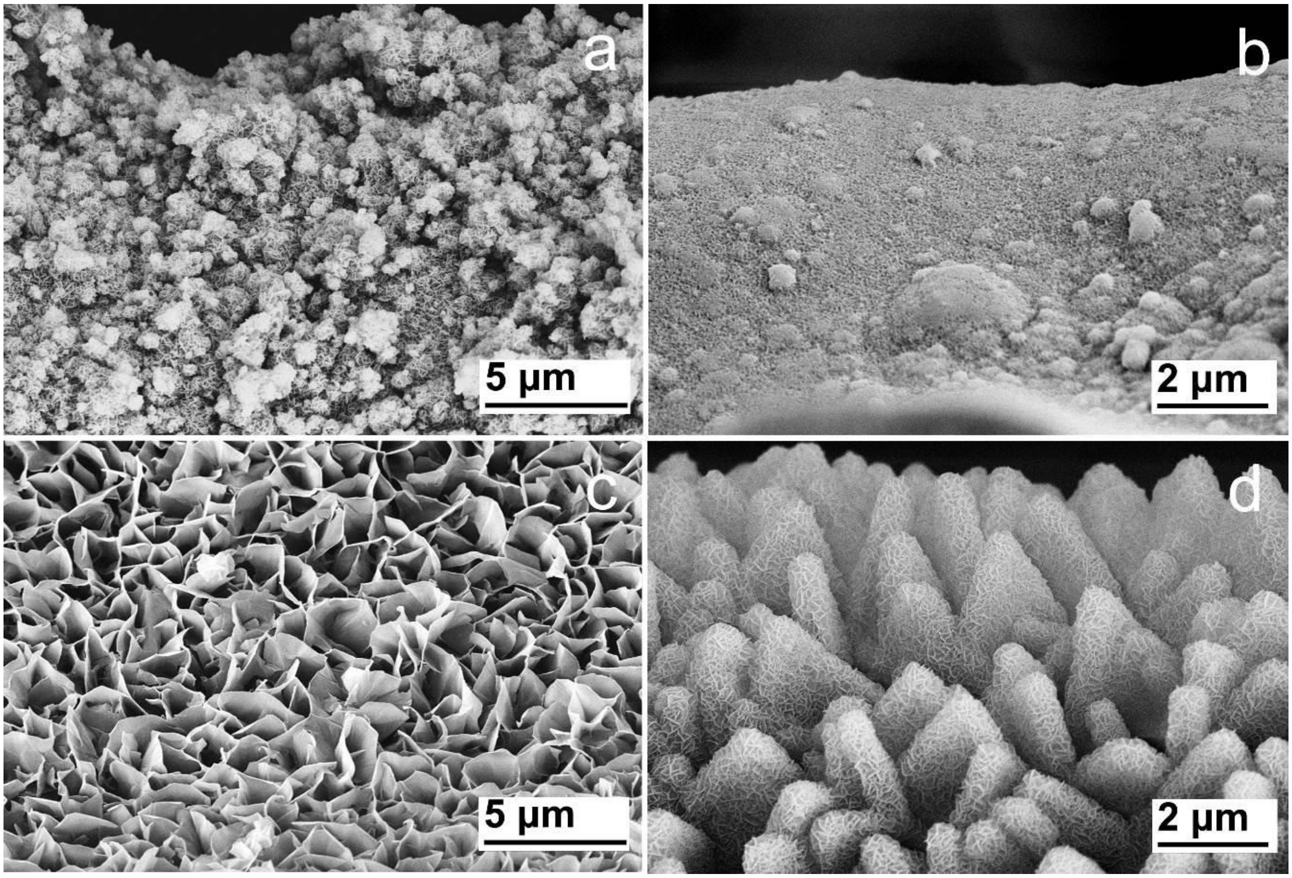

Figure S2. Comparisons of SEM images of (a) NiCo hydroxide, (b) $Ni(OH)_2$, (c) $Co(OH)_2$, and (d) NiCo-90.

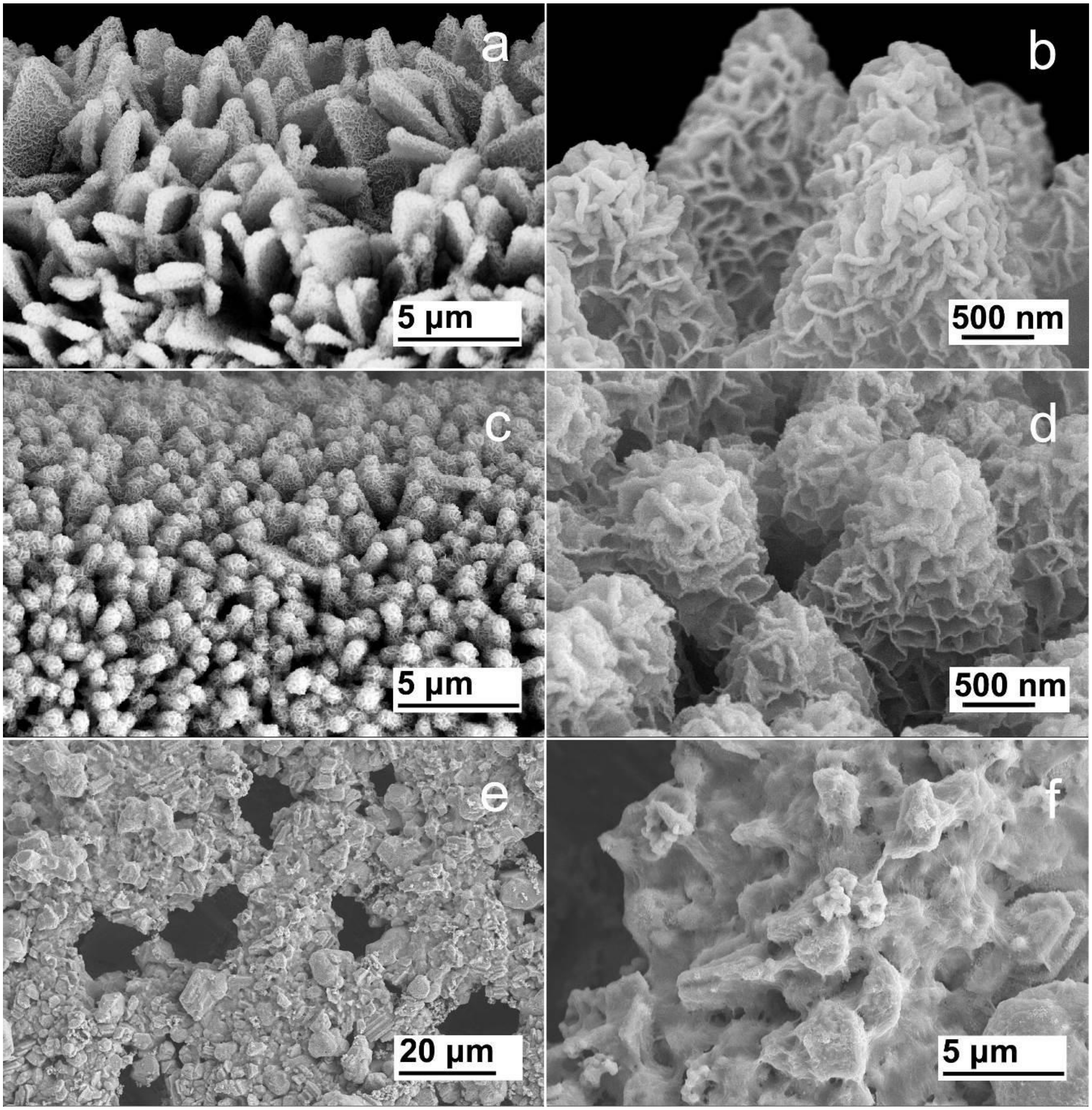

Figure S3. (a, b) SEM images of the NiCo-90 electrode after three charge-discharge cycles at 40 mA cm^{-2}. SEM images of (c, d) NiCo-90 cathode and (e, f) Zn anode after the cycling performance test.

Table S1. Comparison of voltage hysteresis of Ni-Zn batteries reported recently and our NiCo-90//Zn battery.

**Batteries**	**Voltage hysteresis**	**Reference**
CC-CF@NiO//CC-CF@ZnO	0.10 V	[1]
$Co(CO_3)_{0.5}(OH)_x \cdot 0.11H_2O$@$CoMoO_4$//3D CC-ZnO@C-Zn	0.10 V	[2]
Ni–NiO//Zn	0.10 V	[3]
Ni_3S_2//Zn	0.10 V	[4]
Co_3O_4//Zn	0.10 V	[5]
$NiCo_2O_4$//Zn	0.10 V	[6]
P–$NiCo_2O_{4-x}$//Zn	0.12 V	[7]
NiO-CNT//Zn	0.12 V	[8]
Ni_2P/C//Zn	0.12 V	[9]
Ni@NiO//Zn	0.20 V	[10]
**NiCo-90//Zn**	**0.068 V**	**This work**

References for supporting information

[1] J. Liu, C. Guan, C. Zhou, Z. Fan, Q. Ke, G. Zhang, C. Liu, J. Wang, *Adv. Mater.* **2016**, *28*, 8732.

[2] M. Li, J. Meng, Q. Li, M. Huang, X. Liu, K. A. Owusu, Z. Liu, L. Mai, *Adv. Funct. Mater.* **2018**, *28*, 1802016.

[3] Y. Zeng, Y. Meng, Z. Lai, X. Zhang, M. Yu, P. Fang, M. Wu, Y. Tong, X. Lu, *Adv. Mater.* **2017**, *29*, 1702698.

[4] P. Hu, T. Wang, J. Zhao, C. Zhang, J. Ma, H. Du, X. Wang, G. Cui, *ACS Appl. Mater. Interfaces* **2015**, *7*, 26396.

[5] X. Wang, F. Wang, L. Wang, M. Li, Y. Wang, B. Chen, Y. Zhu, L. Fu, L. Zha, L. Zhang, Y. Wu, W. Huang, *Adv. Mater.* **2016**, *28*, 4904.

[6] J. Wang, Z. Jia, S. Li, Y. Wang, W. Guo, T. Qi, *B. Mater. Sci.* **2015**, *38*, 1435.

[7] Y. Zeng, Z. Lai, Y. Han, H. Zhang, S. Xie, X. Lu, *Adv. Mater.* **2018**, *30*, 1802396.

[8] X. Wang, M. Li, Y. Wang, B. Chen, Y. Zhu, Y. Wu, *J. Mater. Chem. A* **2015**, *3*, 8280.

[9] J. Li, C. Chen, *Mater. Res. Express* **2018**, *5*, 015502.

[10] R. Wang, Y. Han, Z. Wang, J. Jiang, Y. Tong, X. Lu, *Adv. Funct. Mater.* **2018**, *28*, 1802157.
